# Founder mutations characterise the mutation panorama in 200 Swedish index cases referred for Long QT syndrome genetic testing

**DOI:** 10.1186/1471-2261-12-95

**Published:** 2012-10-25

**Authors:** Eva-Lena Stattin, Ida Maria Boström, Annika Winbo, Kristina Cederquist, Jenni Jonasson, Björn-Anders Jonsson, Ulla-Britt Diamant, Steen M Jensen, Annika Rydberg, Anna Norberg

**Affiliations:** 1 Department of Medical Biosciences, Medical and Clinical Genetics, Umeå University, Umeå, Sweden; 2 Department of Clinical Sciences, Paediatrics, Umeå University, Umeå, Sweden; 3 Heart Centre and Department of Public Health and Clinical Medicine, Umeå University, Umeå, Sweden

**Keywords:** Arrhythmia, Long QT syndrome, Ion-channel, Founder mutation, Variant of unknown significance

## Abstract

**Background:**

Long QT syndrome (LQTS) is an inherited arrhythmic disorder characterised by prolongation of the QT interval on ECG, presence of syncope and sudden death. The symptoms in LQTS patients are highly variable, and genotype influences the clinical course. This study aims to report the spectrum of LQTS mutations in a Swedish cohort.

**Methods:**

Between March 2006 and October 2009, two hundred, unrelated index cases were referred to the Department of Clinical Genetics, Umeå University Hospital, Sweden, for LQTS genetic testing. We scanned five of the LQTS-susceptibility genes (*KCNQ1, KCNH2, SCN5A, KCNE1*, and *KCNE2*) for mutations by DHPLC and/or sequencing. We applied MLPA to detect large deletions or duplications in the *KCNQ1, KCNH2, SCN5A, KCNE1,* and *KCNE2* genes. Furthermore, the gene *RYR2* was screened in 36 selected LQTS genotype-negative patients to detect cases with the clinically overlapping disease catecholaminergic polymorphic ventricular tachycardia (CPVT).

**Results:**

In total, a disease-causing mutation was identified in 103 of the 200 (52%) index cases. Of these, altered exon copy numbers in the *KCNH2* gene accounted for 2% of the mutations, whereas a *RYR2* mutation accounted for 3% of the mutations. The genotype-positive cases stemmed from 64 distinct mutations, of which 28% were novel to this cohort. The majority of the distinct mutations were found in a single case (80%), whereas 20% of the mutations were observed more than once. Two founder mutations, *KCNQ1* p.Y111C and *KCNQ1* p.R518*, accounted for 25% of the genotype-positive index cases. Genetic cascade screening of 481 relatives to the 103 index cases with an identified mutation revealed 41% mutation carriers who were at risk of cardiac events such as syncope or sudden unexpected death.

**Conclusion:**

In this cohort of Swedish index cases with suspected LQTS, a disease-causing mutation was identified in 52% of the referred patients. Copy number variations explained 2% of the mutations and 3 of 36 selected cases (8%) harboured a mutation in the *RYR2* gene. The mutation panorama is characterised by founder mutations (25%), even so, this cohort increases the amount of known LQTS-associated mutations, as approximately one-third (28%) of the detected mutations were unique.

## Background

Long QT syndrome (LQTS) is an autosomal dominant inherited arrhythmogenic disease and a significant cause of sudden cardiac death (SCD), usually in young and otherwise healthy individuals. LQTS is characterised by delayed ventricular repolarisation, seen as prolongation of the QT-interval on the electrocardiogram (ECG), which predisposes to Torsade-de-Pointes (TdP) and subsequent sudden death by ventricular fibrillation [1,2]. TdP or ventricular tachyarrhythmia is often self-terminating and presents as syncope with loss of consciousness, the most frequent symptom of LQTS. The phenotype is highly variable in expressivity and incomplete in penetrance [3]. Although the majority of LQTS patients show a diagnostic prolongation of the QT-interval on resting ECG, a normal ECG with a normal QTc is not enough to rule out LQTS, since up to approximately 40% of the patients may present with a normal QT-interval. A LQTS mutation carrier without prolonged QTc has a 10% risk of major cardiac events by the age of 40 years when left without treatment [4]. Cardiac events are often prompted by physical activity or by intense emotion or stress, but can also occur at rest or during sleep [5,6]. Exercise-induced syncope can also be caused by another inherited ion-channel disease, named catecholaminergic polymorphic ventricular tachycardia (CPVT), which is characterised by cardiac electrical instability exacerbated by acute activation of the adrenergic nervous system [7]. Some patients suspected to have LQTS might actually have CPVT, since there is a clinical overlap between these disorders [8,9].

The prevalence of LQTS has been estimated to 1/2,000 in the population [10]. To date, 13 different genes have been associated with LQTS, all encoding subunits of cardiac ion-channels (K^+^, Na^+^ or Ca^2+^) or ion-channel regulatory proteins [11]. More than 90% of the mutations are found in five of the genes (*KCNQ1, KCNH2, SCN5A, KCNE1* and *KCNE2*), and mutation analysis of these five LQTS-causing genes reveals a mutation in about 75% of patients with a clinical diagnosis of LQTS [8,12-15]. Typically, the disease-causing mutation is a missense mutation that is unique for the family, although founder mutations have been described in different, relatively isolated populations [16-18]. The occurrence of families with compound heterozygote mutations or apparent digenic inheritance, as well as rare variants of uncertain significance (VUS), further complicates the genetics of LQTS [19,20].

First-degree relatives of a mutation carrier are at 50% risk of carrying the mutation [15], and familial cascade screening should thus be offered immediately to all families with a disease-causing mutation. Identifying additional family members at risk for the condition is of critical importance since they can get preventive treatment, thus decreasing the risk of fatal cardiac events. Here, we examine the spectrum of mutations in 200 unrelated cases referred for LQTS genetic testing in a Swedish population.

## Methods

### Study participants

Between March 2006 and October 2009, a total of 200 unrelated index cases (138 females; 62 males) were referred to the Department of Clinical Genetics, Umeå University Hospital, Sweden, for LQTS genetic testing as part of ordinary health care. Clinical data, including 12-lead ECG, personal history of syncope, treatment with beta-blockers, and family history, was retrospectively collected from referring physicians. The mean age of the 200 index patients at the time of ascertainment was 33 (± 20) years. Corrected QT measurements were obtained from 125 index cases by two different investigators (UBD, SJ) who were blinded to genetic status and the identity of the patient. QT interval was obtained from 12-lead ECG and corrected for heart rate using Bazett’s formula. Additionally, 12 QTc measurements of index cases were obtained from referring clinicians.

For the interpretation of novel missense variants, clinical data and blood samples from family members (both parents and siblings when available) were collected to look for co-segregation between the sequence variant and the disease in the family. Pedigrees were constructed using Cyrillic 2.1 (Cyrillic Software, Oxfordshire, United Kingdom).

The Regional Ethical Review Board of Umeå University approved this study. Data for continuous variables are presented as mean, standard deviations (SD) and/or range. The Mann–Whitney test was used for comparison of QTc, and parametric tests were used for comparison of normally distributed variables. Statistical analysis was performed using GraphPad Prism 5.0 (GraphPad Software, Inc. USA).

### Mutation analysis

DNA was extracted from peripheral blood lymphocytes using a standard salting-out method. Genomic DNA of the 200 index cases were analysed for mutations in all protein-coding exons and their flanking splice site regions of the genes *KCNQ1* (NM_000218.2 and NM_181798.1), *KCNH2* (NM_000238.2 and NM_172057.1), *SCN5A* (NM_198056.1), *KCNE1* (NM_000219.2), and *KCNE2* (NM_172201.1) using PCR, denaturing high-performance liquid chromatography (DHPLC; WAVE, Transgenomic, Omaha, Neb), and/or bi-directional sequencing on the CEQ 8000 (Beckman Coulter, Fullerton, CA, USA) or the ABI 3100 Genetic Analyzer (Applied Biosystems, Foster City, CA, USA). All primers were checked for absence of SNPs to avoid problems with allelic dropout. All five common LQTS genes were analysed regardless of whether a mutation had been identified in one of the genes. Briefly, samples were PCR-amplified by standard methods (primer sequences available on request) and then analysed by DHPLC at one or more temperatures based on the melting profile of the fragment, as determined by Navigator Software version 2.1.0 (Transgenomic). Chromatograms were subjectively grouped, depending on the differences in the profile from normal and known polymorphic variants. Where abnormal patterns of elution were identified, the fragments were sequenced for detection of rare variants. To ensure relevance, all likely pathogenic changes were re-amplified using a different dilution of the same sample.

For cascade screening of relatives, mutation analysis was performed by sequencing as described above, or by directed mutation analysis with MGB probes using the TaqMan 7000 (Applied Biosystems, Foster City, CA, USA). In the latter case, a positive and a genotype- negative familial control was included in each analysis.

All samples were analysed for large deletions or duplications using multiplex ligation-dependent probe amplification (MLPA) with the SALSA P114-A2 kit (MRC-Holland), which covers exons 1B, 1–4, 6–13 and 15–16 for *KCNQ1* (NM_000218.2 and NM_181798.1), exons 1B, 1–4, 6, 9–10 and 14 for *KCNH2* (NM_000238.2 and NM_172057.1), exons 1–2 for *KCNE2* (NM_172201.1), exons 2–4 for *KCNE1* (NM_000219.2), and exons 2, 4, 25 and 27 for *SCN5A* (NM_198056.1). Thirty-six of the LQTS genotype-negative index cases were also analysed for mutations in 23 of the 105 functionally most important exons (8–15, 44–50, 83, 88–105) of the gene *RYR2* (NM_001035.2). These cases were selected for *RYR2* screening based on a history of sudden unexpected death (n=1), aborted cardiac arrest (n=10), ICD treatment (n=3), documented arrhythmia (n=3), and/or syncope (n=31) and/or a family history of SCD (n=11).

### Defining mutation status

Sequences were evaluated using the software Sequencher™ 4.9 (Gene Codes Corporation, MI, USA). All identified LQTS-associated mutations and other variants were denoted using nomenclature recommended by Human Genome Variation Society (HGVS) [21]. To be considered as a LQTS-causing mutation, the variant must disrupt or change either the open reading frame (i.e., missense, nonsense, insertion/deletion, or frame shift mutations) or the conserved splice recognition sequences (the first two intronic nucleotides flanking the exon). Variants that did not change the open reading frame (i.e. synonymous) and intronic variants located outside of the splice recognition sequence (i.e. beyond IVS-2 or IVS+2) were not considered unless an effect on splicing could be predicted using bioinformatic tools (SpliceSiteFinder, MaxEntScan, NNSPLICE and GeneSplicer).

In addition, variants that were previously described in healthy individuals and in NCBI dbSNP as common or rare single nucleotide polymorphisms, such as *KCNQ1* p.P448R, *KCNE1* p.D85N, *SCN5A* p.H558R, or *SCN5A* p.A572D, were not considered to be a pathogenic mutation. *In silico* predictions were made for all putative mutations using the Alamut software version 1.5 (Interactive Biosoftware, Rouen, France). The Alamut software assists in evaluation of missense variants by compiling output from a number of bioinformatic prediction tools, including Polymorphism Phenotyping (PolyPhen), Sorting Intolerant From Tolerant (SIFT) and Align Grantham Variation and Grantham Deviation (Align-GVGD) [22-24].

Novel missense variants were considered mutations only if an effect on the protein could be predicted using bioinformatic tools, and/or if they were absent from 100 control individuals, from the same population, and/or if they co-segregated with affected family members. For all novel missense variants, phylogenetic conservation was evaluated using the Alamut software.

## Results

### Subjects and clinical phenotype

DNA for mutation screening was available from 200 index patients (138 females; 62 male) referred for molecular genetic screening of long QT-syndrome to the Department of Clinical Genetics, Umeå University Hospital, Sweden. Referrals came from all six health care regions, 44 cases from the North health care region, 36 from Uppsala-Örebro, 54 from Stockholm, 43 from the West, 17 from the Southeast, and 6 cases from the South health care region. Questionnaires with clinical data were received for 125 of the index patients from referring physicians. The demographics (age, sex, QTc, symptoms, family history, and treatment with beta-blockers) of all available patients are summarised in Table 1.

**Table 1:** Demographics of all available, unrelated index cases referred for molecular genetic testing regarding Long QT syndrome in ordinary health care

		**Mutation positive**							
	**Total cohort**	***KCNQ1*positive**	***KCNH2*positive**	***SCN5A*positive**	***KCNE1*positive**	***KCNE2*positive**	***RYR2*positive**	**LQTS positive**	**Genotype negative**
Number of index cases	200	60	25	13	1	1	3	100	97
Mean age, SD range, years	33±20 0-79	36±23 0-79	29±17 3-69	24±16 0-52	49	60	20 13-32	34±21 0-79	32±20 0-76
Sex, female/male	138/62	45/15	19/6	6/7	1/0	1/0	1/2	73/27	65/32
Average QTc, SD range, ms	463±44 305-640 (136)	479±37 403-597 (41)	472±30 436-565 (20)	505±66 434-640 (10)	447-(1)	478-(1)	428 395-454 (3)	481±41 403-640 (73)	445±41 305-510 (60)
Syncope, % Yes/No	61% 72/46 (118)	53% 19/17 (36)	68% 13/6 (19)	4/4 (8)	No (1)	No (1)	1/1 (2)	55% 36/29 (65)	69% 35/16 (51)
Family history, % Yes/No	43% 49/65 (114)	76% 29/9 (38)	63% 12/7 (19)	3/3 (6)	Yes (1)	No (1)	1/1 (2)	69% 45/20 (65)	6% 3/44 (47)
Β-blockers, % Yes/No	52% 59/54 (113)	67% 22/11 (33)	68% 13/6 (19)	3/8 (8)	Yes (1)	Yes (1)	2/0 (2)	65% 40/22 (62)	35% 17/32 (49)

The numbers in parenthesis refers to the number of cases in each category. No percentages are calculated if there are less than 10 cases.

**Figure 1:**
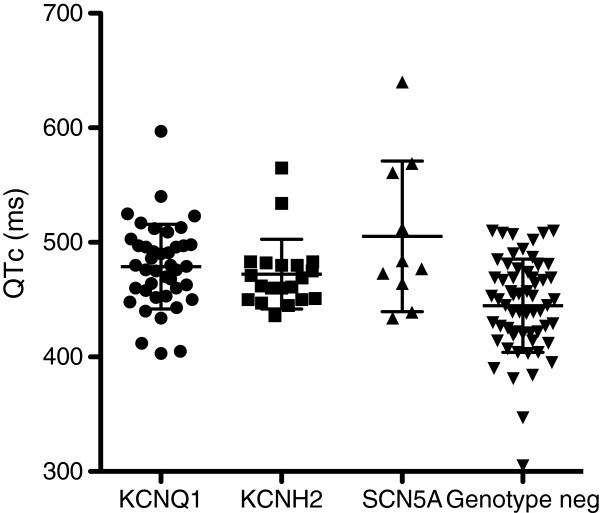
**Graphic illustration of the difference between QTc among mutation carriers (*KCNQ1, KCNH2 *and *SCN5A *) compared with index cases in which no mutation was identified.** The figure shows a significant QTc difference between LQTS mutation carriers and index cases without an identified mutation (*KCNQ1* (n= 41) versus genotype-negative (n=61) p= 0.0001, *KCNH2* (n=20) versus genotype-negative p=0.01, *SCN5A* (n=10) versus genotype-negative p=0.005). The scatter dot plot show QTc mean and SD.

The average age at ascertainment was 33 ±20 years (range 13 days to 79 years). An ECG was available for 137 (68%) of the index cases. The average QTc among all available index patients was 463 ms (± 44). The average QTc was 479 ms (± 37) among *KCNQ1* mutation carriers, 472 ms (± 30) among *KCNH2* mutation carriers, and 505 ms (± 66) among *SCN5A* mutation carriers. The average QTc among index cases without an identified mutation was 445 ms (± 41) (Table 1). Although there was no significant difference in QTc-interval between LQT1-3 mutation carriers, there was a significant difference in QTc interval between mutation carriers and index cases without an identified mutation (Figure 1). Among the genotype-positive cases there were 33 individuals with a QTc ≥480 ms, and among the genotype-negative cases, 12 had QTc ≥480 ms. Among the patients with a prolonged QT interval, 77% carried a LQTS mutation whereas 23% had no mutation. Furthermore, 38% of the patients with a normal QT interval carried a mutation, whereas 62% had no mutation. Among subjects selected for *RYR2* screening (n=36) the average QTc was 432 ms (range 347–477 ms), of which five of them had a QTc prolongation >460 ms (25 available ECG).

### Molecular genetic analysis

In total, a mutation was identified in 103 of the 200 (52%) index patients (Table 2).

**Table 2:** **Pathogenic mutations in the *KCNQ1, KCNE1, KCNH2, KCNE2, SCN5A *and *RYR2 *-genes among Swedish index cases referred for genetic testing with respect to LQTS**

**Gene**	**Exon**	**Nucleotide change**	**Amino acid change**	**Mutation type**	**Region**	**No. of probands**	**Reference**
***KCNQ1***	1	c.217C>A	p.P73T	Missense	N-term	1^b^	Kapplinger *et al.* 2009
	1	c.332A>G	p.Y111C	Missense	N-term	20^b^	Splawski *et al.* 2000
	3	c.506C>G	p.T169R	Missense	S2	1^c^	This study^a^
	3	c.509A>G	p.E170G	Missense	S2-S3	1	This study^a^
	3	c.572_576del	p.R192Cfs91*	Frame shift	S2-S3	3	Tyson *et al.* 1997
	4	c.643G>A	p.V215M	Missense	S3	1	Napolitano *et al.* 2005
	4	c.674C>T	p.S225L	Missense	S4	2	Priori *et al* 1999
	5	c.727C>T	p.R243C	Missense	S4-S5	2	Franqueza *et al.*1999
	5	c.734G>T	p.G245V	Missense	S4-S5	1	This study^a^
	7	c.935C>T	p.T312I	Missense	Pore	1	Wang *et al.* 1996
	7	c.944A>G	p.Y315C	Missense	Pore	1	Splawski *et al.* 1998
	7	c.973G>T	p.G325W	Missense	S6	1	This study^a^
	7	c.973G>A	p.G325R	Missense	S6	2	Tanaka *et al.* 1997
	7	c.1023_1024delinsTT	p.L342F	Missense	S6	1	Donger *et al.* 1997
	7	c.1031C>A	p.A344E	Missense	S6	1	Tester *et al.* 2005
	8	c.1033-1G>C	splice	Splice site	S6	1	This study^a^
	8	c.1046C>G	p.S349W	Missense	C-term	1	Splawski *et al.* 2000
	8	c.1066_1071del	p.Q356_Q357del	Deletion	C-term	1	Liang *et al.* 2003
	10	c.1265delA	p.K422Sfs*10	Frame shift	C-term	1	Kapplinger *et al.* 2009
	12	c.1552C>T	p.R518*	Nonsense	C-term	6	Wei *et al.* 2000
	12	c.1588C>T	p.Q530*	Nonsense	C-term	3	Tranebjærg *et al.* 1999
	13	c.1615C>T	p.R539W	Missense	C-term	1	Chouabe *et al.*1997
	13	c.1664G>A	p.R555H	Missense	C-term	1	Lupoglazoff *et al.* 2004
	14	c.1697C>T	p.S566F	Missense	C-term	1	Splawski *et al.* 2000
	15	c.1766G>A	p.G589D	Missense	SAR	1	Piippo *et al.* 2001
	15	c.1772G>A	p.R591H	Missense	SAR	1	Neyroud *et al.* 1999
	15	c.1780C>T	p.R594*	Nonsense	SAR	1	This study^a^
	15	c.1781G>A	p.R594Q	Missense	SAR	1	Splawski *et al.* 2000
	16	c.1801C>T	p.Q601*	Nonsense	SAR	1	This study^a^
	16	c.1893dup	p.R632Qfs*20	Frame shift	C-term	1^b^	Neyroud *et al.* 1999
***KCNH2***	2	exon 2 duplication		Duplication	N-term	1	This study^a^
	2	c.128A>G	p.Y43C	Missense	PAS	1	Napolitano *et al.* 2005
	2	c.157G>A	p.G53S	Missense	PAS	1	Nagaoka *et al.* 2008
	2	c.182A>G	p.Q61R	Missense	PAS	1^c^	This study^a^
	2	c.235_242del	p.A79Dfs*63	Frame shift	N-term	1	This study^a^
	2	c.244_252dup	p.I82_Q84dup	Insertion	PAC	1	Larsen *et al.* 2001
	2	c.284A>G	p.E95G	Missense	PAC	1^c^	This study^a^
	3	c.453delC	p.T152Pfs*14	Frame shift	N-term	2	Swan *et al.* 1999
	4	c.526C>T	p.R176W	Missense	N-term	1	Swan *et al.* 1999
	4	c.853_859dup	p.D287Gfs*47	Frame shift	N-term	1	This study^a^
	5	c.982C>T	p.R328C	Missense	N-term	1	Tester *et al.* 2005
	5	c.1094A>G	p.E365G	Missense	N-term	1	This study^a^
	7	c.1655T>C	p.L552S	Missense	S5	2	Swan *et al.* 1999
	7	c.1688G>A	p.W563*	Nonsense	S5	1	Berge *et al.* 2005
	7	c.1706A>G	p.Y569C	Missense	S5	1	This study^a^
	7	c. 1750G>A	p.G584S	Missense	S5	1	Swan *et al.* 1999
	9	c.2254C>T	p.R752W	Missense	cNBD	1	Splawski *et al.* 2000
	9	c.2312A>G	p.H771R	Missense	cNBD	1	This study^a^
	10	c.2453C>T	p.S818L	Missense	cNBD	1	Berthet *et al.* 1999
	9-10	exon 9-10 deletion		Deletion	cNBD/C-term	1	This study^a^
	12	c.2959_2960del	p.L987Vfs*131	Frame shift	C-term	2	Splawski *et al.* 2000
	13	c.3107dupG	p.D1037Rfs*82	Frame shift	C-term	1	Berthet *et al.* 1999
***SCN5A***	2	c.86C>T	p.A29V	Missense	N-term	1^b^	This study^a^
	7	c.715A>G	p.I239V	Missense	DI-S4/S5	1	Fodstad *et al.* 2004
	10	c.1231G>A	p.V411M	Missense	DI-S6	4^bc^	Tester *et al.* 2005
	22	c.3893C>T	p.P1298L	Missense	DIII-S4	1	This study^a^
	23	c.4000A>G	p.I1334V	Missense	DIII-S4/S5	1	Kapplinger *et al.* 2009
	26	c.4519_4527del	p.Q1507_P1509del	Deletion	DIII-DIV	4	Keller *et al.* 2003
	28	c.4877G>C	p.R1626P	Missense	DIV-S4	1	Napolitano *et al.* 2005
	28	c.5350G>A	p.E1784K	Missense	C-term	1	Wei *et al.* 1999
***KCNE1***	4	c.95G>A	p.R32H	Missense	Extracellular	1^b^	Splawski *et al.* 2000
***KCNE2***	2	c.170T>C	p.I57T	Missense	Transmembrane	1	Abbott *et al.* 1999
***RYR2***	44	c.6737C>T	p.S2246L	Missense	Cytoplasmatic loop	2^c^	Priori *et al.* 2001
	101	c.14553C>A	p.F4851L	Missense	TM domain	1	Hayashi *et al.* 2009

^a^ Denotes a novel variant, unique to this cohort. ^b^ Compound heterozygous or homozygous mutations ^c^*de novo* mutation.

Sequence analysis of the LQTS-associated genes revealed a pathogenic mutation in 98 of the 200 index patients. Furthermore, the MLPA analysis revealed 2 different CNVs in *KCNH2* (exon 2 dup, and exon 9–10 del) in two patients, and the *RYR2* screening revealed a pathogenic mutation in 3 of the 36 selected patients (8%). Mutations in the *KCNQ1* gene were most prevalent (58%), followed by *KCNH2* (24%), *SCN5A* (13%), *RYR2* (3%), *KCNE1* (1%) and *KCNE2* (1%). Among the 103 mutation-positive patients, 99 had a single heterozygous mutation, whereas four female patients (4%) carried multiple mutations (*KCNQ1* c.1893dup; QTc 512 ms, *KCNE1* p.R32H; QTc 447 ms, *KCNQ1* p.P73T, *SCN5A* p.V411M; QTc 464 ms and *KCNQ1* p.Y111C, *SCN5A* p.A29V; QTc 490ms); two homozygous and two compound heterozygous mutation carriers (Table 2). None of the patients hosting double mutations displayed the phenotype of Jervell and Lange-Nielsen syndrome with clinical deafness, even though the mutations resided on different alleles.

The 103 genotype-positive patients stemmed from 64 distinct mutations, the majority of which were observed in a single case (n=51). Of the 13 mutations that were observed more than once, the six most common were *KCNQ1* p.Y111C (n=20), *KCNQ1* p.R518* (n=6), *SCN5A* p.V411M (n=4), *SCN5A* c.4519_4527del (n=4), *KCNQ1* p.Q530* (n=3), and *KCNQ1* c.572_576del (n=3).

Approximately one-third (28%) of the mutations had never previously been reported in LQTS at the time of detection, and were thus novel to this Swedish cohort. These include seven mutations in *KCNQ1*, nine in *KCNH2* and two in *SCN5A* (Table 2). None of these mutations were observed in 100 analysed population-matched control individuals. Furthermore, with the exception of *SCN5A* p.P1298L, none were present in any of the large whole exome sequencing projects “1000 genomes project” or “NHBLI exome sequencing project (ESP)”. The putative mutation *SCN5A* p.P1298L was referred to as rs28937319 in dbSNP with unknown allele frequency and status probable-pathogenic. Although it has been associated with sick sinus syndrome [25], it has never been reported previously in LQTS patients, and is thus considered a novel LQTS mutation.

Evaluation of the pathogenicity in seven of the novel mutations was straightforward, including two stop mutations (*KCNQ1* p.R594*, and *KCNQ1* p.Q601*), two frame-shift mutations resulting in premature stops (*KCNH2* p.A79Dfs*63, and *KCNH2* p.D287Gfs*47), two CNVs in *KCNH2* as described above, and one splice mutation in position IVS-1 (*KCNQ1* c.1033-1G>C). For the eleven novel missense variants, the most important factor in determining the pathogenicity was the degree to which a missense change was conserved in orthologs and in other proteins with the same domain (Table 3). Phylogenetic alignments for the novel missense mutations are presented in Additional file 1, showing that the mutations affected highly conserved residues. *In silico* data and co-segregation are summarised in Table 3. *In silico* analysis using SIFT predicted that none of the variants seen would be tolerated, which is consistent with them being pathogenic [22]. PolyPhen conservation scores predicted seven of the variants to be probably damaging and four to be possibly damaging (Table 3) [23]. In addition, co-segregation analyses of the novel missense variants were performed in all families where samples from relatives were available. The pedigrees showed perfect co-segregation between the novel sequence variant and the disease in six families, whereas in four families no interpretation was possible due to non-penetrant or borderline QTc, and in five other families there was no samples available or missing data (data not shown). In the remaining three families, the variant had occurred *de novo*.

**Table 3:** Characteristics of the novel missense mutations unique to the Swedish cohort

**Gene**	**Exon**	**Nucleotide change**	**Amino acid change**	**Region**	**GD**	**SIFT**	**PolyPhen**	**Align-GVGD**	**Segregation analysis**
***KCNQ1***	3	c.506C>G	p.T169R	S2	71	not tolerated	Possibly damaging	C0	*de novo*
	3	c.509A>G	p.E170G	S2-S3	98	not tolerated	Probably damaging	C0	Yes
	5	c.734G>T	p.G245V	S4-S5	109	not tolerated	Probably damaging	C0	Borderline
	7	c.973G>T	p.G325W	S6	184	not tolerated	Probably damaging	C65	Yes
***KCNH2***	2	c.182A>G	p.Q61R	PAS	43	not tolerated	Possibly damaging	C0	*de novo*
	2	c.284A>G	p.E95G	PAC	98	not tolerated	Probably damaging	C0	*de novo*
	5	c.1094A>G	p.E365G	N-term	98	not tolerated	Possibly damaging	C0	Yes
	7	c.1706A>G	p.Y569C	S5	194	not tolerated	Probably damaging	C65	Borderline
	9	c.2312A>G	p.H771R	cNBD	29	not tolerated	Probably damaging	C25	N/A
***SCN5A***	2	c.86C>T	p.A29V	N-term	65	not tolerated	Probably damaging	C65	Yes
	22	c.3893C>T	p.P1298L	DIII-S4	98	not tolerated	Possibly damaging	C65	N/A

GD, Grantham distance ordered from largest difference (GD=215) between the substituted amino acids to no difference (GD=0); SIFT, sorting intolerant from tolerant; PolyPhen, Polymorphism Phenotyping predicting variants as probably damaging, possibly damaging or benign; Align-GVGD, Align Grantham variation and Grantham distance ordered from most likely (C65) to interfere with function to least likely (C0); Segregation analysis: Yes, segregation demonstrated; *de novo*, mutation not present in either parent; Borderline, non-penetrant or borderline QTc; N/A, samples not available or missing data.

Among the 64 distinct mutations, missense mutations were most common (70%), followed by frame-shift mutations (12.5%), nonsense mutations (8%), in-frame deletions/insertions (5%), large deletions/insertions (3%), and splice-site mutations (1.5%) (Table 4). Most of the 64 distinct mutations (47%) were localised to the transmembrane spanning and pore-forming domains, whereas 25% were localised to the N-terminus, and 28% to the C-terminus.

**Table 4:** Summary of population screening studies of long QT syndrome

		Splawskiet al. 2000	Tester et al. 2005 Tester et al. 2005^a^	Napolitano et al. 2005	Berge et al. 2008	Kapplinger et al. 2009	**This study**
Number of unrelated index cases (n)		262	541	430	169	2500	200
Detection rate (%) All cases/more stringent criteria (*Schwartz score ≥ 4)		51	50/72*	72	32/71	36	52
Novel mutations (%)		60	59	59	54	60	28
Multiple mutations (%)		-	10	5	0	9	4
Mutated gene:	*KCNQ1* (%)	39	42	49	43	43	58
	*KCNH2* (%)	51	42	39	46	32	24
	*SCN5A* (%)	6	15	10	9	13	13
	*KCNE1* (%)	2	0.5	2	2	3	1
	*KCNE2* (%)	2	0.5	1	-	1	1
	*RYR2* (%)	-	- 269/6^a^	-	- 41/17	-	3 36/8
	*RYR2* (n/%)						
Mutation type:	Missense (%)	72	73	72	65	70	70
	Nonsense (%)	6	6	5	14	6	8
	In-frame ins/del (%)	5	2	14	3	3	5
	Frame shift (%)	10	12	6	13	15	12.5
	Splice site (%)	7	6	3	5	6	1.5
	Large ins/del (%)	-	-	-	-	-	3
Mutation region:	N-terminal (%)	22	16	8	22	8	25
	Transmembrane (%)	54	49	64	54	57	47
	C-terminal (%)	24	35	28	24	35	28

In 97 of the 200 index patients, no pathogenic mutation was identified. In 19 of these patients rare missense variants were detected, that could potentially contribute to the disease phenotype, but these were not considered to be pathogenic by themselves. Additional file 2 describes all identified rare variants in the Swedish cohort with a frequency less than 5%, as well as all missense substitutions, all of which were classified as normal genetic variants.

### Genetic cascade screening in family members

In the 103 unrelated families where a LQTS or CPVT disease-causing mutation was identified, a total of 481 relatives have undergone cascade genetic testing. Of these relatives, 199 (41%) were mutation carriers, while 282 (59%) did not carry a mutation. The mean number of tested individuals in each family was 5.7 (the proband included). In total, 5 of the 105 (5%) identified mutations had occurred *de novo* in the index patient (four LQTS mutations and one CPVT mutation, *KCNQ1* p.T169R, *KCNH2* p.E95G, *KCNH2* p.Q61R, *SCN5A* p.V411M, and *RYR2* p.S2246L, see Table 2). However, since both parents were not tested in all families, it is possible that this number is higher (data not shown). In one of the families with a *de novo* mutation (*SCN5A p.*V411M), two children but none of the parents were carriers, indicating possible germ-line mosaicism.

## Discussion

In this study, we have determined the mutation panorama in a Swedish cohort referred for genetic LQTS testing as part of ordinary health care. Between March 2006 and October 2009, the department of Clinical Genetics in Umeå was to our knowledge the only laboratory in Sweden screening the LQTS genes. Among the 200 index patients, 64 different mutations were identified in 103 patients (52%); of which 58% occurred in *KCNQ1*, 24% in *KCNH2*, 13% in *SCN5A*, 3% in *RYR2*, 1% in *KCNE1*, and 1% in *KCNE2*. Thirteen of the mutations were found in more than one family, whereas 51 occurred only once. Among these mutations, 28% were novel at the time of detection, and had thus never been reported previously.

### LQTS founder mutations

Two of the recurring mutations, *KCNQ1* p.Y111C and *KCNQ1* p.R518*, were identified in 26 of the 103 cases, thus accounting for approximately 25% of the mutations in the Swedish LQTS population. We have recently shown that family members carrying these mutations share a common haplotype that is specific for each mutation [26] [Abstract number 154:Winbo A. Stattin E.L. Nordin C. Persson J. Diamant U.B. Jensen S.M. Rydberg A. Origin, genotype and clinical phenotype of the Long QT Syndrome R518X/KCNQ1 mutation in Sweden. Presented at the 46th Annual Meeting of the Association for European Paediatric and Congenital Cardiology (AEPC), May 23–26 2012 in Istanbul]. The mutation *KCNQ1* p.Y111C was introduced and enriched in the Ångerman River valley approximately 600 years ago [26]. Functional *in-vitro* studies have demonstrated that it is a “malignant” mutation with a strong dominant-negative effect, causing disturbed function of the wild-type ion channel [27,28]. In contrast to these findings, we recently showed that the *KCNQ1* p.Y111C mutation presents with a low incidence of life threatening events in a Swedish Y111C-positive LQTS population [29]. Furthermore, we showed that p.Y111C is a founder mutation in this population [26], a finding which also contrasts to *in vitro*-data indicating it to be a malignant mutation. One explanation for these discrepancies could be the presence of population-specific modifiers, genetic or other, such as the recently described polymorphisms in the 3’-UTR of *KCNQ1*, mitigating the effect of the mutated allele by reduced expression [30]. Possibly, one or several of these polymorphisms could exert its attenuating effect through the creation of a novel miRNA-binding site, a theory that has been proposed for other disorders where large differences in phenotypic expression occur [31].

The mutation *KCNQ1* p.R518* has previously been reported in several populations [32,33], as well as a founder mutation in Sweden [34] and Norway [16], although Berge *et al*. did not report any founder mutations in the more recent Norwegian LQTS population survey [8]. A strong founder effect has been described in the Finnish population [17]. In our study, we identified three of the Finnish founder mutations (*KCNQ1* p.G589D, *KCNH2* p.R176W, and *KCNH2* p.L552S), as well as the two common Norwegian mutations (*KCNQ1* p.R518* mentioned above, and *KCNQ1* p.Q530*) in several of the patients [16,35].

### LQTS genotype-negative index cases

According to published studies, approximately 25% of index cases with the clinical phenotype of LQTS remain genotype-negative after comprehensive assessment of the three most common LQTS genes (*KCNQ1, KCNH2,* and *SCN5A*) [12,13,15]. In this study, 102 index patients (51%) referred for LQTS testing were negative after sequencing of the *KCNQ1, KCNH2, KCNE1*, *KCNE2* and *SCN5A* genes. As in any molecular genetic study of disease, there is a possibility that these individuals have mutations missed due to technical limitations (e.g. DHPLC), or located in regions not included in the analysis; such as in the gene promotors or introns of the genes chosen for study, or in another LQTS-associated gene. However, these patients had a significantly shorter QTc, and reported family history than the mutation carriers, and some of them might therefore be suspected of not having LQTS (Figure 1, Table 1). Several publications indicate that patients suspected of having LQTS may actually have CPVT, [8,9,36] and that can be confirmed also in the present study. Among 36 genotype-negative index patients selected for *RYR2* screening based on a history of arrhythmia, aborted cardiac arrest and/or syncope and/or a family history of SCD, we identified a disease-causing mutation in 8%. Tester *et al*. evaluated the prevalence of *RYR2* mutations in a cohort of patients referred for screening of LQTS genes, identifying mutations in *RYR2* among 6% of the 269 genotype-negative patients [9]. Berge *et al.* identified mutations in *RYR2* in 17% of the 41 genotype-negative index patients referred for LQTS testing [8]. Thus, it is critical to recognise CPVT as an important differential diagnosis to LQTS, and to consider mutation screening of the *RYR2* gene in patients who do not have a mutation in one of the LQTS-associated genes.

The presence of copy number variants (CNVs) within the LQTS disease genes have also been suggested as an explanation for the lack of identified mutations [37-39]. Therefore, we performed MLPA analysis of all 200 index patients, identifying 2 CNVs in the *KCNH2* gene. Thus, the yield of CNVs was 2.0% among the 100 genotype-negative index patients without an identified mutation in any of the LQTS genes or *RYR2*. In the study of Tester *et al*. CNVs were found in 4.8% of 42 patients with QTc duration ≥ 480 ms and/or a Schwartz score ≥ 4 who were negative for mutations in 12 of the LQTS-associated genes [40]. Eddy *et al.* identified CNVs in 11.5% of 26 patients with Schwartz score ≥ 4 who were negative for mutations in the *KCNQ1, KCNH2*, and *SCN5A* genes [38]. Barc *et al*. identified CNVs in 3.2% of 93 patients with Schwartz score ≥ 3 who were negative for mutations in the *KCNQ1, KCNH2*, and *SCN5A* genes [39]. These findings suggest that CNVs might be a more frequent cause of LQTS than mutations in all of the less common LQTS-associated genes (*ANKB, KCNE1, KCNE2, KCNJ2, CACNA1C, CAV3, SCN4B, AKAP9*, and *SNTA1)* together [38-40]. Thus, it is important to consider MLPA analysis in patients who do not possess a mutation in one of the most common LQTS-associated genes.

### Genetic cascade screening in family members

A total of 481 relatives in the 103 families with an identified mutation have participated in genetic cascade screening, of which 41% were found to be mutation carriers, and 59% were not carriers. Thus, 2.9 (302/103) individuals per family carried a mutation and were thereby at risk for LQTS-associated symptoms and SCD. This finding is lower than the result in Norway, where, 4.7 (305/66) patients per family carried a heterozygote mutation [8]. In contrast to Imboden et al. [41] no female predominance among mutation carriers and no non-random inheritance, with a significant greater number of affected than expected, could be observed in this cohort.

### This study compared with other population surveys

In the five largest LQTS population surveys that have been published to date, involving the five most common LQTS-causing genes (*KCNQ1, KCNH2, SCN5A, KCNE1* and *KCNE2*), the mutation yield was 72%, 51%, 50%, 36%, and 32%, respectively (Table 4) [8,12-15]. In two of the studies, when using more stringent criteria (i.e. Schwartz score ≥ 4), the mutation detection rate was raised from 50% to 72%, and from 32% to 71%, respectively [8,13]. In this study, we obtained a mutation detection rate of 52%, which lies in the range of the other population studies. Among individuals with a definite prolonged QTc, 77% carried a mutation, which is in line with the two studies using more stringent criteria. We were not able to categorise all the index patients, since phenotypic information was not available for all of the patients.

The largest survey, including 2,500 consecutive, unrelated LQTS patients, presented one of the lowest mutation yields of 36%. However, the degree of diagnostic relevance in the referred patients of that study could not be evaluated, also due to lack of phenotypic information.

The mutations in the *KCNQ1, KCNH2* and *SCN5A* genes were distributed over the entire coding regions and adjacent splice sites. The vast majority were heterozygous missense mutations. The distribution of mutations between the different genes and the type of mutation concur with findings of the other population surveys. However, the rate of *KCNQ1* mutations is higher in our study (58%), since both of the Swedish founder mutations p.Y111C and p.R518* are located in this gene. Most of the mutations (≈60 %) in the published surveys have not been reported previously, whereas we only identified 28% novel mutations in this study. It is possible that this lower yield is due to the more than 10 years of publications of several large LQTS studies, which suggests that the increase in new LQTS mutations is beginning to be saturated.

### Probands carrying multiple mutations

Patients carrying multiple mutations have been shown to present with a more severe phenotype compared to patients carrying only one mutation [19]. In one study, the compound mutation carriers had longer QTc intervals and a younger age-at-onset compared to patients with only one mutation [19]. In this study, four of the 103 (4%) genotype-positive patients carried more than one definitely pathogenic mutation. Two of them were homozygous for the mutation and two compound heterozygote. All of them are female and have a family history of LQTS. The (homozygous) carrier of the *KCNQ1* c.1893dup mutation, had a QTc prolongation of 512 ms; however, there is no information about any history of syncope or current treatment with beta-blockers. The parents of the c.1893dup mutation carrier both carried the mutation in heterozygous form; the mother had a QTc of 435 ms while the father has a QTc denoted as normal (data not shown). The *KCNE1* p.R32H (homozygous) mutation carrier, had a QTc of 447 ms, is being treated with beta-blockers and has not experienced syncope. The parents of the p.R32H mutation carrier were not available for testing, but hemizygosity of the mutation in the proband was excluded by MLPA (data not shown). The carrier of the (compound heterozygote) mutations *KCNQ1* p.P73T and *SCN5A* p.V411M, had a history of suspected seizures and syncope, and she was not treated with beta-blockers but with Phenytoin. The carrier of the (compound heterozygote) mutations *KCNQ1* p.Y111C and *SCN5A* p.A29V, had a history of presyncope and a QTc of 490 ms.

In the Norwegian survey, no patients had more than one definitely pathogenic mutation, whereas Kapplinger *et al*. reported 9%, and Tester *et al*. reported 11% patients with multiple mutations among the genotype-positive patients [8,13,14]. In the study of Westenskow *et al.* compound mutations were reported in 12% of the genotype-positive LQTS probands. However, of the 20 probands in their study assigned as having multiple mutations, over half possessed either the *KCNE1* p.D85N or *KCNQ1* p.P448R common polymorphism as the “second hit” [42]. Similarly, Tester *et al.* reported *SCN5A* p.A572D as a mutation in 3 of their patients [13]. In our study, *SCN5A* p.A572D was identified in 3 patients, *KCNQ1* p.P448R in 3 patients, and *KCNE1* p.D85N in 12 patients, all of which were determined to be non-pathogenic ( Additional file 2). In four of the patients, these variants occurred together with a definitely pathogenic mutation. If we had regarded these as pathogenic mutations, our yield of multiple mutations among the genotype-positive patients would have been 8% (8/103) instead of 4%.

### Sequence variants of unknown significance - polymorphisms

Missense mutations are the most frequent form of mutation in the LQTS genes, accounting for about 65-73% of the mutations in the large LQTS population surveys. Careful interpretation of identified genetic variants is important, because a missense variant may or may not cause an altered/distorted protein and a disease phenotype [43]. In this study, several of the index patients carried rare variants, such as *KCNE1* p.D85N, *KCNQ1* p.P448R, *SCN5A* p.A572D, *SCN5A* p.S1103Y, and *SCN5A* p.R1193Q. The possible effect of these missense variants is difficult to interpret and they are referred to in the literature as both mutations and functional polymorphisms [44,45]. Although these variants might contribute to the phenotype, we did not consider these as disease-causing mutations by themselves, since careful interpretation of genetic test results is critical in clinical practice [43].

#### Limitations of the study

The 200 index cases were almost consecutively included; we have excluded 27 index cases with other diagnoses such as Jervell and Lange-Nielsen syndrome, Brugada Syndrome, short QT syndrome, and healthy individuals sent for LQTS-screening due to a history of first-degree relative with SUD. No selection of the patients was performed, resulting in a cohort that ranges from low suspicion of LQTS to high. Since the patients were referred for LQTS screening in ordinary health care, the clinical data were collected retrospectively and is thus not complete for all families. Only 23 of 105 exons (8–15, 44–50, 83, 88–105) of the gene *RYR2* were analysed*.* DNA was not available for screening of all five genes in some of the individuals, and therefore it is possible that some double mutations might have been missed. Due to the lack of DNA, there were incomplete analyses of the *KCNQ1* gene in three individuals, in the *KCNH2* gene in two, *KCNE1* in five, *KCNE2* gene in four, and in the *SCN5A* gene in nine individuals*.* For the same reason MLPA was not performed in two cases.

## Conclusions

The distribution of mutations between the different genes, as well as the type of mutation, concur with findings of other LQTS population surveys. In contrast, the mutation panorama in this Swedish cohort is characterised by two founder mutations in the *KCNQ1* gene that accounts for one-fourth of the identified mutations. The findings of a mutation in *RYR2* among 8% of the selected cases, as well as CNVs among 2% of all genotype-negative cases suggest that mutation analysis of *RYR2* and MLPA analysis in a genotype-negative LQTS population is of importance and might give a higher yield than screening of the less common LQTS-associated genes.

## Abbreviations

CNV: Copy Number Variations; CPVT: Catecholaminergic Polymorphic Ventricular Tachycardia; DHPLC: Denaturing High-Performance Liquid Chromatography; DNA: Deoxyribonucleic Acid, ECG, Electrocardiogram; HGVS: Human Genome Variation Society; LQTS: Long QT syndrome; MLPA: Multiplex Ligation-dependent Probe Amplification; PCR: Polymerase Chain Reaction; SCD: Sudden Cardiac Death; TdP: Torsade-de-Pointes; VUS: Variants of Uncertain Significance.

## Competing interests

The authors declare that they have no competing interests.

## Authors’ contributions

ELS: conception and design, acquisition of data, analysis, interpretation of data, drafting the manuscript. IMB: carried out most of the molecular genetic studies, analysis, interpretation of data and revision of manuscript. KC, JJ: analysis, interpretation of data and revision of manuscript. BAJ: analysis, interpretation of data. AW, UBD: analysis and revision of manuscript. AN, SJ, AR: conception and design, analysis and revision of manuscript. All authors read and approved the final manuscript.

## Pre-publication history

The pre-publication history for this paper can be accessed here:

http://www.biomedcentral.com/1471-2261/12/95/prepub

## Supplementary Material

Additional file 1 **Phylogenetic alignments for eleven novel mutations identified in the*KCNQ1, KCNH2,*and*SCN5A*genes in 200 index cases referred for genetic screening with respect to LQTS in a Swedish cohort.** Click here for file

Additional file 2 **Non-pathogenic variants in the *KCNQ1, KCNH2, SCN5A, KCNE1, *and *KCNE2 *genes. ** Variants within *RYR2* are not reported due to small sample size. All missense substitutions, rare silent substitutions (MAF less than 5%) and variants within −5 or +5 from the exon boundary are reported. Common silent substitutions (MAF more than 5%) and intronic variants more than 5 bp from an exon/intron boundary are not reported. NA = frequency of data not available. Click here for file
