# Supplementary material for: Founder mutations characterise the mutation panorama in 200 Swedish index cases referred for Long QT syndrome genetic testing
Source: BMC Cardiovasc Disord. 2012 Oct 25;12:95. doi: 10.1186/1471-2261-12-95 (PMC3520728; doi:10.1186/1471-2261-12-95)
Supplement: Additional file 1 — Phylogenetic alignments for eleven novel mutations identified in theKCNQ1, KCNH2,andSCN5Agenes in 200 index cases referred for genetic screening with respect to LQTS in a Swedish cohort. [file 1471-2261-12-95-S1.pdf]

KCNQ1

```
p.T169R             LERPTGWKCFVYHFAVFLIVLVCLIFSVLSTIEQYAALATGTLFWMEIVLVVFFGREYVV 173
Human               LERPTGWKCFVYHFAVFLIVLVCLIFSVLSTIEQYAALATGTLFWMEIVLVVFFGTEYVV 173
Chimpanzee          LERPTGWKCFVYHFAVFLIVLVCLIFSVLSTIEQYAALATGTLFWMEIVLVVFFGTEYVV 173
Maqaque             LERPTGWKCFVYHFAVFLIVLVCLIFSVLSTIEQYAALATGTLFWMEIVLVVFFGTEYVV 140
Rat                 LERPTGWKCFVYHFTVFLIVLVCLIFSVLSTIEQYAALATGTLFWMEIVLVVFFGTEYVV 173
Mouse               LERPTGWKCFVYHFTVFLIVLVCLIFSVLSTIEQYAALATGTLFWMEIVLVVFFGTEYVV 172
Dog                 ---------------------------------------------QEIVLVVFFGTEYVV 15
Cow                 ----------------------------------------------EIVLVVFFGTEYVV 14
Opossum             ------------------------------------------------------------
Chicken             LERPTGWKCFVYHFTVFLIVLICLIFSVLSTIEQYAALATGTLFWMEIVLVVFFGTEYVV 176
Western_clawed_frog LKKPLSWKPFVYYTPKFLIVLICLIFSVLSTIQQYNNLATETLFWMEIVLVVFFGAEYVV 64
Pufferfish          LYKEG------TQSSRFLIVLACLILSVLSTIDQYQSLSQTTLFWVEIVLVVFFGMEYFV 61
```

```
p.E170G             LERPTGWKCFVYHFAVFLIVLVCLIFSVLSTIEQYAALATGTLFWMEIVLVVFFGTGYVV 173
Human               LERPTGWKCFVYHFAVFLIVLVCLIFSVLSTIEQYAALATGTLFWMEIVLVVFFGTEYVV 173
Chimpanzee          LERPTGWKCFVYHFAVFLIVLVCLIFSVLSTIEQYAALATGTLFWMEIVLVVFFGTEYVV 173
Maqaque             LERPTGWKCFVYHFAVFLIVLVCLIFSVLSTIEQYAALATGTLFWMEIVLVVFFGTEYVV 140
Rat                 LERPTGWKCFVYHFTVFLIVLVCLIFSVLSTIEQYAALATGTLFWMEIVLVVFFGTEYVV 173
Mouse               LERPTGWKCFVYHFTVFLIVLVCLIFSVLSTIEQYAALATGTLFWMEIVLVVFFGTEYVV 172
Dog                 ---------------------------------------------QEIVLVVFFGTEYVV 15
Cow                 ----------------------------------------------EIVLVVFFGTEYVV 14
Opossum             ------------------------------------------------------------
Chicken             LERPTGWKCFVYHFTVFLIVLICLIFSVLSTIEQYAALATGTLFWMEIVLVVFFGTEYVV 176
Western_clawed_frog LKKPLSWKPFVYYTPKFLIVLICLIFSVLSTIQQYNNLATETLFWMEIVLVVFFGAEYVV 64
Pufferfish          LYKEG------TQSSRFLIVLACLILSVLSTIDQYQSLSQTTLFWVEIVLVVFFGMEYFV 61
```

```
p.G245V             QILRMLHVDRQVGTWRLLGSVVFIHRQELITTLYIGFLGLIFSSYFVYLAEKDAVNESGR 293
Human               QILRMLHVDRQGGTWRLLGSVVFIHRQELITTLYIGFLGLIFSSYFVYLAEKDAVNESGR 293
Chimpanzee          QILRMLHVDRQGGTWRLLGSVVFIHRQELITTLYIGFLGLIFSSYFVYLAEKDAVNESGR 293
Maqaque             QILRMLHVDRQGGTWRLLGSVVFIHRQELITTLYIGFLGLIFSSYFVYLAEKDAVNESGR 260
Rat                 QILRMLHVDRQGGTWRLLGSVVFIHRQELITTLYIGFLGLIFSSYFVYLAEKDAVNESGR 293
Mouse               QILRMLHVDRQGGTWRLLGSVVFIHRQELITTLYIGFLGLIFSSYFVYLAEKDAVNESGR 292
Dog                 QILRMLHVDRQGGTWRLLGSVVFIHRQELITTLYIGFLGLIFSSYFVYLAEKDAVNDSGQ 135
Cow                 QILRMLHVDRQGGTWRLLGSVVFIHRQELITTLYIGFLGLIFSSYFVYLAEKDAVNESGQ 134
Opossum             ------------------------------------------------------------
Chicken             QILRMLHVDRQGGTWRLLGSVVFIHRQELITTLYIGFLGLIFSSYFVYLAEKDAVNDSGE 296
Western_clawed_frog QILRMLHVDRQGGTWRLLGSVVFIHRQELITTLYIGFLGLIFSSYFVYLAEKDAIDSSGE 184
Pufferfish          QILRMLHVDRQGGTWRLLGSVVYIHRQELITTLYIGFLSLIFSSYFVYLAETYSVS-GGS 180
```

```
p.G325W             VEFGSYADALWWGVVTVTTIGYGDKVPQTWVWKTIASCFSVFAISFFALPAGILGSGFAL 353
Human               VEFGSYADALWWGVVTVTTIGYGDKVPQTWVGKTIASCFSVFAISFFALPAGILGSGFAL 353
Chimpanzee          VEFGSYADALWWGVVTVTTIGYGDKVPQTWVGKTIASCFSVFAISFFALPA--------- 344
Maqaque             VEFGSYADALWWGVVTVTTIGYGDKVPQTWVGKTIASCFSVFAISFFALPAGILGSGFAL 320
Rat                 IEFGSYADALWWGVVTVTTIGYGDKVPQTWVGKTIASCFSVFAISFFALPAGILGSGFAL 353
Mouse               IEFGSYADALWWGVVTVTTIGYGDKVPQTWVGKTIASCFSVFAISFFALPAGILGSGFAL 352
Dog                 VEFGSYADALWWGVVTVTTIGYGDKVPQTWVGKTIASCFSVFAISFFALPAGILGSGFAL 195
Cow                 VEFGSYADALWWGVVTVTTIGYGDKVPQTWVGKTIASCFSVFAISFFALPAGILGSGFAL 194
Opossum             --------------VTVTTIGYGDKVPQTWIGKTIASCFSVFAISFFALPAGILGSGFAL 46
Chicken             TEFGSYADALWWGVVTVTTIGYGDKVPQTWIGKTIASCFSVFAISFFALPAGILGSGFAL 356
Western_clawed_frog YQFGSYADALWWGVVTVTTIGYGDKVPQTWIGKTIASCFSVFAISFFALPAGILGSGFAL 244
Pufferfish          TDFRNFADALWWGVVTVTTIGYGDKVPQTWIGKTIASCFSVFAISFFALPAGILGSGFAL 240
                                  ****************: *******************
```

KCNH2

```
p.Q61R                        GFCELCGYSRAEVMRRPCTCDFLHGPRTQRRAAAQIAQALLG------AE 90
Human                         GFCELCGYSRAEVMQRPCTCDFLHGPRTQRRAAAQIAQALLG------AE 90
Chimpanzee                    GFCELCGYSRAEVMQRPCTCDFLHGPRTQRRAAAQIAQALLG------AE 90
Rhesus_macaque                GFCELCGYSRAEVMQRPCTCDFLHGPRTQRRAAAQIAQALLG------AE 90
Brown_rat                     GFCELCGYSRAEVMQRPCTCDFLHGPRTQRRAAAQIAQALLG------AE 90
House_mouse                   GFCELCGYSRAEVMQRPCTCDFLHGPRTQRRAAAQIAQALLG------AE 90
Dog                           GSWGRCGGGSLSRPSVYPSAAAADTPCLHPPRLASAPGAEGAGGRLQASQ 96
Cattle                        GFCELCGYSRAEVMQRPCTCDFLHGPRTQRRAAAQIAQALLG------AE 64
Gray_short-tailed_opossum     GFCELCGYSRAEVMQRPCTCDFLHGPRTQRRAAAQIAQALLG------AE 94
Western_clawed_frog           --------------------------------------------------
Zebrafish                     ----MCGYTRAEIMQKPCTCNFLYGPHTKRLAIAQMAQALLG------SE 40
```

```
p.E95G                        ERKVGIAFYRKDGSCFLCLVDVVPVKNEDGAVIMFILNFEVVMEKDMVGS 140
Human                         ERKVEIAFYRKDGSCFLCLVDVVPVKNEDGAVIMFILNFEVVMEKDMVGS 140
Chimpanzee                    ERKVEIAFYRKDGSCFLCLVDVVPVKNEDGAVIMFILNFEVVMEKDMVGS 140
Rhesus_macaque                ERKVEIAFYRKDGSCFLCLVDVVPVKNEDGAVIMFILNFEVVMEKDMVGS 140
Brown_rat                     ERKVEIAFYRKDGSCFLCLVDVVPVKNEDGAVIMFILNFEVVMEKDMVGS 140
House_mouse                   ERKVEIAFYRKDGSCFLCLVDVVPVKNEDGAVIMFILNFEVVMEKDMVGS 140
Dog                           ERKVEIAFYRKDGSCFLCLVDVVPVKNEDGAVIMFILNFEVVMEKDMVGS 146
Cattle                        ERKVEIAFYRKDGSCFLCLVDVVPVKNEDGAVIMFILNFEVVMEKGLVGS 114
Gray_short-tailed_opossum     ERKVEIAFYRKDGSCFLCLVDVVPVKNEDGAVIMFILNFEVVMETELTGS 144
Western_clawed_frog           --------------------------------------------------
Zebrafish                     ERKVEIALYRKDGVCFLCLVDVVPVKNEDGVVIMFILNFEVMPDDKLHDP 90
```

```
p.E365G                       SKIPQITLNFVDLKGDPFLASPTSDREIIAP-KIKGRTHNVTEKVTQVLS 379
Human                         SKIPQITLNFVDLKGDPFLASPTSDREIIAP-KIKERTHNVTEKVTQVLS 379
Chimpanzee                    SKIPQITLNFVDLKGDPFLASPTSDREIIAP-KIKERTHNVTEKVTQVLS 379
Rhesus_macaque                SKIPQITLNFVDLKGDPFLASPTSDREIIAP-KIKERTHNVTEKVTQVLS 378
Brown_rat                     SKIPQITLNFVDLKGDPFLASPTSDREIIAP-KIKERTHNVTEKVTQVLS 381
House_mouse                   SKIPQITLNFVDLKGDPFLASPTSDREIIAP-KIKERTHNVTEKVTQVLS 381
Dog                           SKIPQITLNFVDLKGDPFLASPTSDREIIAP-KIKERTHNVTEKVTQVLS 384
Cattle                        SKIPQITLNFVDLKGDPFLASPTSDREIIAP-KIKERTHNVTEKVTQVLS 323
Gray_short-tailed_opossum     SKIPQITLNFVDLKGDPFLASPASDREIIAP-KIKERTHNVTEKVTQVLS 381
Western_clawed_frog           ------------------LCLP------------------------QVLS 8
Zebrafish                     SKIPQITLNFVDFKPDPLIALPAGEMDIIAPCKLIDRTHNVTEKVTQVLS 336
                                                :. *                        ****
```

```
p.Y569C                       IGLLKTARLLRLVRVARKLDRYSEYGAAVLFLLMCTFALIAHWLACIWCA 570
Human                         IGLLKTARLLRLVRVARKLDRYSEYGAAVLFLLMCTFALIAHWLACIWYA 570
Chimpanzee                    IGLLKTARLLRLVRVARKLDRYSEYGAAVLFLLMCTFALIAHWLACIWYA 570
Rhesus_macaque                IGLLKTARLLRLVRVARKLDRYSEYGAAVLFLLMCTFALIAHWLACIWYA 569
Brown_rat                     IGLLKTARLLRLVRVARKLDRYSEYGAAVLFLLMCTFALIAHWLACIWYA 572
House_mouse                   IGLLKTARLLRLVRVARKLDRYSEYGAAVLFLLMCTFALIAHWLACIWYA 572
Dog                           IGLLKTARLLRLVRVARKLDRYSEYGAAVLFLLMCTFALIAHWLACIWYA 575
Cattle                        IGLLKTARLLRLVRVARKLDRYSEYGAAVLFLLMCTFALIAHWLACIWYA 514
Gray_short-tailed_opossum     IGLLKTARLLRLVRVARKLDRYSEYGAAVLFLLMCTFALIAHWLACIWYA 570
Western_clawed_frog           IGLLKTARLLRLVRVARKLDRYSEYGAAVLFLLMCTFALIAHWLACIWYA 201
Zebrafish                     IGLLKTARLLRLVRVARKLDRYSEYGAAVLFLLMCTFALIAHWLACIWYA 535
                              ************************************************ *
```

```
p.H771R                         TLVRAGDLLTALYFISRGSIEILRGDVVVAILGKNDIFGEPLNLYARPGK 817
Human                           TLVHAGDLLTALYFISRGSIEILRGDVVVAILGKNDIFGEPLNLYARPGK 817
Chimpanzee                      TLVHAGDLLTALYFISRGSIEILRGDVVVAILGKNDIFGEPLNLYARPGK 817
Rhesus_macaque                  TLVHAGDLLTALYFISRGSIEILRGDVVVAILGKNDIFGEPLNLYARPGK 816
Brown_rat                       TLVHAGDLLTALYFISRGSIEILRGDVVVAILGKNDIFGEPLNLYARPGK 819
House_mouse                     TLVHAGDLLTALYFISRGSIEILRGDVVVAILGKNDIFGEPLNLYARPGK 819
Dog                             TLVHAGDLLTALYFISRGSIEILRGDVVVAILGKNDIFGEPLNLYARPGK 822
Cattle                          TLVHAGDLLTALYFISRGSIEILRGDVVVAILGKNDIFGEPLNLYARPGK 761
Gray_short-tailed_opossum       TLVHAGDLLTALYFISRGSIEILRGDVVVAILGKNDIFGEPLNLYARPGK 817
Western_clawed_frog             TLVHAGDVLTAVYFLSRGSIEILRGDVVVAILGKNDIFGEPINLYARPGK 450
Zebrafish                       --------------------------------------------------
```

SCN5A

```
p.A29V              MANFLLPRGTSSFRRFTRESLAAIEKRMVEKQARG-STTLQESREGLPEEEAPRPQLDLQ 59
Human               MANFLLPRGTSSFRRFTRESLAAIEKRMAEKQARG-STTLQESREGLPEEEAPRPQLDLQ 59
Chimpanzee          MANFLLPRGTSSFRRFTRESLAAIEKRMAEKQARG-STTLQESREGLPEEEAPRPQLDLQ 59
House_Mouse         MANFLLPRGTSSFRRFTRESLAAIEKRMAEKQARG-SATSQESREGLPEEEAPRPQLDLQ 59
Brown_rat           MANLLLPRGTSSFRRFTRESLAAIEKRMAEKQARGGSATSQESREGLQEEEAPRPQLDLQ 60
Cattle              MAAFLLPRGTSSFRRFTRESLAAIEKRMAEKQARS-SAASQESRDGLPEEEAPRPQLDLQ 59
Horse               MADFLLPRGTSSFRRFTRESLAAIEKRMAEKQARG-LAASQESREGLPEEEAPRPQLDLQ 59
Dog                 MADFLLPRGTSSFRRFTRESLAAIEKRMAEKQARG-SAASQESREGLPEEEAPRPQLDLQ 59
Red_jungle_fowl     MADFLLPPGTNSFHRFTPESLAAIEKRIAEKLARN---AKQEYREQLGEEEKPQPQFDLQ 57
Zebrafish           MAAILFPPGPDSLHRFTRESLAGIEQRIAEEEARN----AKRYQEDRGDVEPPKPRADLE 56
                    ** :*:* *..*::*** ****.**:*:.*: **.     :. ::   : * *:*: **:
```

```
p.P1298L            VANTLGFAEMGLIKSLRTLRALRPLRALSRFEGMRVVVNALVGAIPSIMNVLLVCLIFWL 1346
Human               VANTLGFAEMGPIKSLRTLRALRPLRALSRFEGMRVVVNALVGAIPSIMNVLLVCLIFWL 1346
Chimpanzee          VANTLGFAEMGPIKSLRTLRALRPLRALSRFEGMRVVVNALVGAIPSIMNVLLVCLIFWL 1346
House_Mouse         VANTLGFAEMGPIKSLRTLRALRPLRALSRFEGMRVVVNALVGAIPSIMNVLLVCLIFWL 1348
Brown_rat           VANTLGFAEMGPIKSLRTLRALRPLRALSRFEGMRVVVNALVGAIPSIMNVLLVCLIFWL 1348
Cattle              VANALGFAEMGPIKSLRTLRALRPLRALSRFEGMRVVVNALVGAIPSIMNVLLVCLIFWL 1351
Horse               VANTLGFAEMGPIKSLRTLRALRPLRALSRFEGMRVVVNALVGAIPSIMNVLLVCLIFWL 1349
Dog                 VANTLGFAEMGPIKSLRTLRALRPLRALSRFEGMRVVVNALVGAIPSIMNVLLVCLIFWL 1343
Red_jungle_fowl     IANTLGYSEMGPIKSLRTLRALRPLRALSRFEGMRVVVNALVGAIPSIMNVLLVCLIFWL 1361
Zebrafish           VANTLGYSDFAAIKSLRTLRALRPLRALSRFEGMRVVVNALIGAIPSIMNVLLVCLIFWL 1289
                    :**:**::::. *****************************:******************
```
