# Supplementary material for: Founder mutations characterise the mutation panorama in 200 Swedish index cases referred for Long QT syndrome genetic testing
Source: BMC Cardiovasc Disord. 2012 Oct 25;12:95. doi: 10.1186/1471-2261-12-95 (PMC3520728; doi:10.1186/1471-2261-12-95)
Supplement: Additional file 2 — Non-pathogenic variants in the KCNQ1, KCNH2, SCN5A, KCNE1, and KCNE2 genes. Variants within RYR2 are not reported due to small sample size. All missense substitutions, rare silent substitutions (MAF less than 5%) and variants within −5 or +5 from the exon boundary are reported. Common silent substitutions (MAF more than 5%) and intronic variants more than 5 bp from an exon/intron boundary are not reported. NA = frequency of data not available. [file 1471-2261-12-95-S2.pdf]

Gene	Exon	Nucleotide change	Amino acid change	rs-number (dbSNP)	Variation type	Index cases (n)	Minor allele frequency (MAF) dbSNP
KCNQ1	1	c.1-5T>C	-	unknown	Intron	1	NA
	5	c.720C>T	p.H240H	rs28730754	Silent	1	T=0.005/11
	10	c.1179G>T	p.K393N	rs12720457	Missense	1	T=0.001/1
	10	c.1343C>G	p.P448R	rs8179001	Missense	3	NA
	16	c.1926C>T	p.C642C	rs12720454	Silent	1	NA
	16	c.1942G>A	p.V648I	rs34150427	Missense	1	A=0.005/12
KCNH2	4	c.638A>G	p.D213G	unknown	Missense	1	NA
	7	c.1812C>T	p.G604G	unknown	Silent	1	NA
	11	c.2690A>C	p.K897T	rs1805123	Missense	56	G=0.129/283
	12	c.2948C>T	p.T983I	rs149955375	Missense	1	NA
	13	c.3140G>T	p.R1047L	rs36210421	Missense	14	A=0.017/36
SCN5A	3	c.354C>T	p.H118H	rs45533640	Silent	2	A=0.001/1
	4	c.462A>T	p.P154P	unknown	Silent	1	NA
	5	c.553G>A	p.A185T	rs192113333	Missense	1	NA
	6	c.630G>A	p.V210V	unknown	Silent	1	NA
	7	c.717C>T	p.I239I	rs41285129	Silent	1	NA
	8	c.993C>T	p.D331D	unknown	Silent	1	NA
	10	c.1141-3C>A	-	rs41312433	Intron	54	T=0.145/318
	12	c.1587T>C	p.I529I	rs45624133	Silent	1	G=0.003/7
	12	c.1653G>A	p.A551A	unknown	Silent	1	NA
	12	c.1673A>G	p.H558R	rs1805124	Missense	66	C=0.206/451
	12	c.1681C>T	p.L561L	rs45522138	Silent	2	A=0.001/2
	12	c.1715C>A	p.A572D	rs36210423	Missense	3	A=0.001/1
	17	c.3093C>T	p.G1031G	unknown	Silent	1	NA
	18	c.3308C>A	p.S1103Y	rs7626962	Missense	1	T=0.012/26
	20	c.3578G>A	p.R1193Q	rs41261344	Missense	1	T=0.011/25
	23	c.4218G>A	p.G1406G	rs41311123	Missense	5	A=0.001/1
	26	c.4509C>T	p.S1503S	rs45548237	Silent	1	A=0.002/5

	28	c.4824C>T	p.L1608L	rs45437099	Silent	1	NA
	28	c.4848C>T	p.F1616F	rs41315495	Silent	1	A=0.034/75
	28	c.5851G>T	p.V1951L	rs41315493	Missense	1	A=0.004/9
	28	c.6010T>C	p.F2004L	rs41311117	Missense	1	G=0.001/3
KCNE1	4	c.29C>T	p.T10M	rs144917638	Missense	1	NA
	4	c.112A>G	p.S38G	rs1805127	Missense	51	NA
	4	c.253G>A	p.D85N	rs1805128	Missense	12	NA
	4	c.293G>A	p.R98Q	rs150454912	Missense	1	NA
KCNE2	2	c.22A>G	p.T8A	rs2234916	Missense	2	NA

Additional file 2. Non-pathogenic variants in the *KCNQ1, KCNH2, SCN5A, KCNE1,* and *KCNE2*-genes. Variants within *RYR2* are not reported due to small sample size. All missense substitutions, rare silent substitutions (MAF less than 5%) and variants within -5 or +5 from the exon boundary are reported. Common silent substitutions (MAF more than 5%) and intronic variants more than 5 bp from an exon/intron boundary are not reported. NA = available.
